# Recent advancements in real-world microbial fuel cell applications

**DOI:** 10.1016/j.coelec.2018.09.006

**Published:** 2018-10

**Authors:** Iwona Gajda, John Greenman, Ioannis A. Ieropoulos

**Affiliations:** 1 Bristol BioEnergy Centre, Bristol Robotics Laboratory, University of the West of England, BS16 1QY, UK; 2 Centre for Research in Biosciences, University of the West of England, BS16 1QY, UK

## Abstract

This short review focuses on the recent developments of the Microbial Fuel Cell (MFC) technology, its scale-up and implementation in real world applications. Microbial Fuel Cells produce (bio)energy from waste streams, which can reduce environmental pollution, but also decrease the cost of the treatment. Although the technology is still considered “new”, it has a long history since its discovery, but it is only now that recent developments have allowed its implementation in real world settings, as a precursor to commercialisation.

## Highlights

• Microbial Fuel Cell (MFC) scale up challenges are discussed.
• Cost benefit analysis discussed.
• New MFC applications, pilot studies and field trials are invaluable tools for further innovation.
• Modular configuration simplifies stack implementation.

## Graphical abstract

**Figure:**
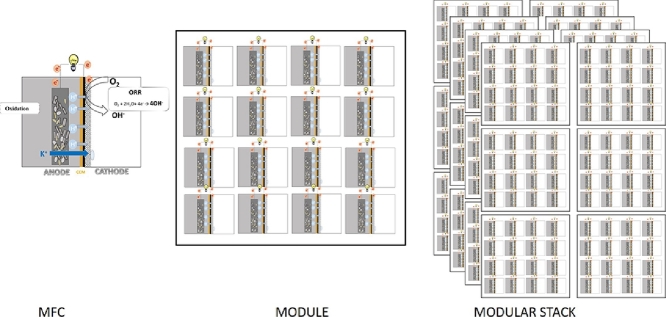

**Current Opinion in Electrochemistry** 2018, **11**:78–83

This review comes from a themed issue on **Environmental Electrochemistry**

Edited by **Nicole Jaffrezic-Renault** and **Christine Mousty**

For a complete overview see the Issue and the Editorial

Available online 02 October 2018

https://doi.org/10.1016/j.coelec.2018.09.006

2451-9103/© 2018 Elsevier B.V. This is an open access article under the CC BY license. (http://creativecommons.org/licenses/by/4.0/)

## Introduction

Although the Microbial Fuel Cell (MFC) technology was firstly presented by Michael Potter in 1911, the knowledge and interest in this type of environmental–focused technology continues to expand. The microbial-derived electrochemistry sprung into Bioelectrochemical Systems (BES) that exploit the process of bioelectrochemical utilisation of organic matter via microbial metabolism, to generate usable by-products, fuels and bio-electricity. BES includes MFCs, Microbial Desalination Cells (MDCs) and Microbial Electrolysis Cells (MECs) amongst others, however it is only the MFC that is purposefully designed to deliver direct electric current from the breakdown of multiple substrates and sources of waste [1]. The technology is versatile as it offers direct power but also feedstock treatment [2], nutrient recovery [3] and sensing [4] for real-time monitoring of processed substrates. The implementation of MFC technology is a promising alternative to the use of classical aerated activated sludge treatment processes [5^•^]. Therefore any pilot study, field trial, or prototype installation is adding a valuable body of knowledge contributing to the technology readiness for real world implementation and a wider market. There is a large body of literature that focusses on laboratory based systems, analytical techniques and improvements achieved from novel materials [6], including non-platinum-group electrocatalysts for improved oxygen-reduction-reaction (ORR) at the cathode [7], structurally and morphologically modified electrode materials [8^•^], as well as hydraulic and electrical stacking of multiple units [9] and power optimisation methods [10^•^]. However pilot studies are uncommon due to the complexity of installation and operating procedures as well as other engineering and environmental factors. Historically in 1931 Barnett Cohen, connected multiple 10 mL bacteria –based fuel cells together in series forming the first MFC stack, generating a total of 2 mA and 35 V [11]. From that time the potential of MFC technology has been widely demonstrated however rarely implemented in practice and real life environments.

As with any prototype development, several key performance factors are investigated for optimisation, which set the agenda for trialling out a particular technology in the field. For MFCs these can be categorised as follows:

## MFC unit design

MFC performance is affected by the reactor architecture and its individual components. However these are all determined by specific application requirements, as MFCs can be used in pilot applications for power production, treatment and sensing; this study is primarily focused on system performance and cost. Developing anode materials suitable for use in microbial fuel cells needs to meet the criteria of high electrical conductivity, high surface area and biocompatibility that would allow for efficient electrochemical “wiring”(attachment) of living bacterial cells [12^•^] but also high conductivity, corrosion resistance and chemical stability with cost as the primary driver. Anode component is critical in terms of the surface area available for the development of the microbial biofilm and for the purpose of meeting all the criteria, carbon based materials either fabric-based such as cloth, mesh, felt etc or granular are usually chosen [13]. Research studies have established that by increasing the anodic surface area to volume ratio 14., 15.• and anode packing 16., 17. would facilitate cost-effective improvement in power density. Apart from enhancing electricity generation this can boost treatment efficiency and degradation of pollutants [17]. The development of new low cost solutions such as stainless steel wool [18] or carbon sponge [19] is a promising anode alternative and requires testing in long term experiments. Optimizing cathode components is based on the reduction reaction occurring in the cathodic half-cell. ORR that is taking place at the MFC cathode which is often the limiting reaction and therefore a source of losses [20^•^]. The most feasible cathode configuration for large-scale application of MFCs is an air-breathing cathode; however it shows limited performance under static operation mode [21]. This is often connected to the cathode scaling with precipitated salts and its deactivation in time 22., 23.. It is then required to configure the cathodic chamber accordingly to avoid salt precipitation. One way is via the development of a catholyte-generating half-cell which provides good long-term performance since the generated liquid washes away the deposits [24] and provides additional electrochemical treatment through disinfection and bacterial killing [25].

## Membrane or membrane-less configuration

Although membrane-less systems can be characterized by less complex design, decreased internal resistance, and lower cost due to the absence of membrane, they nevertheless lose efficiency due to the occurrence of ionic species crossover and side-reactions. Due to simplicity and low cost they can be implemented in water bodies for bioremediation [26] and environmental sensing 27.•, 28.••, or to provide a power source for charging mobile phones [29]. However, for the purpose of the anolyte/catholyte separation a membrane is required and this needs to be chosen appropriately to the application that is then a subject of cost and simplicity of assembly/manufacturing [30]. The choice of robust and low cost membrane materials should be considered to meet the criteria of mechanical strength and longevity under various operating conditions in real world applications.

## Scale

### Multiplication and miniaturisation

Power and current densities significantly decrease with the enlargement of the physical (geometrical) size of the reactor [31]. For example, a module with a total volume of 250 L consisting of two MFC units achieved a relatively low power density of 0.47 W m^3^ [32]. This is because of the increase in the internal resistance in the anodic, cathodic, membrane and electrolyte components. Miniaturisation of MFCs is one direction that allows for increased power densities and can be implemented in MFC stacks 9., 33., 34., 35.. In order to overcome the practical challenges, the reactor should consist of modules involving multiple electrodes and/or multiple MFC units. Division into modules (parts of the whole system), hydraulic isolation of these modules, and multiplication of units is necessary for stepping-up the voltage values, when connected in series, in order to avoid short circuiting [36^•^]. The modularity is here represented by the components of the module (anodes and cathodes) that can be connected electrically in parallel due to the fact that they share the same electrolyte therefore they form a group of multiple MFC units.

### Large-scale studies

The power output generated from an individual MFC unit is insufficient for most practical applications, therefore to increase power, series configuration of individual MFC units needs to be implemented into a stack. There are several ways of testing large-scale prototypes. One way is the integration of the MFC based system with in a wastewater treatment facility. In terms of large scale, pilot studies have recently presented an integration of membrane-less 45-L stack [37^••^] into a municipal wastewater treatment plant that showed stability even when operated on low concentration of Chemical Oxygen Demand (COD). Another study focused on a 72-L stack made of 5 membrane-based MFC units with a power density of 50.9 W/m^3^ however it suffered from electrical current losses in the parallel circuit [38^••^]. A pilot study utilising brewery wastewater of a total volume of 90 L [39^•^] (five 18 L modules) used cloth separator to prevent short-circuiting between the anode and the cathode and used the produced electricity to drive the pumping system. Another modular 200 L implementation treating primary effluent reported by Ge and He was able to reach 75% removal of total chemical oxygen demand using generated electricity to drive the catholyte recirculation pump [40^••^]. Large-scale reactors can include up to 1000-L made of 50 modules achieving up to 90% COD removal and up to 60 W/m^3^ when operated on real municipal wastewater for 1 year [41^••^]. Another study tested low cost MFC system treating brewery wastewater for almost 1 year and showed up to 98% COD removal [42^••^] which might be associated to low flow rate and high hydraulic retention time (HRT).

## Modularity and stacking as a means of scaling up

### Microbial fuel cell in stacks. Electrical and hydraulic connections

Microbial Fuel Cell technology is currently at the laboratory level stage of analysis and evaluation, but some new and ingenious designs have been developed in the recent years to incorporate MFCs into real world implementations. This includes the development of stackable units in order to multiply MFC components. Stacking MFCs can be done for the purpose of the wastewater treatment by connecting multiple units/modules that share a common fuel feed passage in a flow through system. The tubular approach is a viable option and connecting 5 units [43] in a parallel configuration was reaching up to 175.7 W/m^2^ and up to 77% COD removal. However, when the two identical MFCs were connected in series, the systems suffered from an open circuit voltage loss when connected, both electrically (due to the parasitic current flow in the underperforming fuel cell) and hydraulically (due to the internal ionic short-circuiting) 9., 36.•, 46.. Constructing serpentine-like large number of units (40 MFCs) achieved increased voltage up to 23 V however the performance deteriorated in long term operation [45] due to cathode deactivation by salt deposits.

The build-up of salts 43., 46. and biofilm [47] is a common problem in long-term operation, especially when an outer, open to air cathode is employed 43., 44., 46. and it was a major obstacle in the first MFC pilot conducted at Foster's brewery in Yatala, Queensland in 2008 [48^•^]. One way to overcome this is by inverting the design where the cathode is on the inside of the tubular reactor 25., 49.•, 50., 51. protecting it from evaporation or using a partially submerged cathode to keep it sufficiently hydrated [29]. Long term operation is important in terms of the practical reasons including the stability of current generation and internal resistance that are the key issues relevant to the pilot studies.

To achieve high power densities in MFCs, the main obstacle is the system architecture, not the composition or the ecosystem of the anodic community [52]. However, the bacterial community is determined by the type of wastewater that is being processed with a specific organic loading, pH, chemical composition and salinity, as well as electrode material that would also affect the internal resistance of the system. Therefore, the prototype should be designed according to the specific task (sensing/power/treatment) and environment that it will be installed in and the type and flow rate of substrate processed.

## Manufacturing and cost

All of the above have been presented from a basic science lab-based perspective, conducive with earlier technology stage development, under controlled conditions. However, the greatest challenge for any technology moving into the real world, is its suitability for manufacturing, which in turn, drives economies of scale. The same applies to the MFC development and this has been part of the challenge in the technology taking off and becoming commercially available. Most of the core parts and components can be bespoke and therefore expensive, even at prototype level, and there is a scientific challenge in identifying alternatives that would (i) perform equally well and for prolonged periods but most importantly (ii) be inexpensive and widely available. One of the avenues researchers have explored are the alternative low cost materials including ceramic [53,54] or cardboard 55., 56. and plant derived electrodes [57]. As reported by Ge and He in long term operation of their pilot study in wastewater treatment plant, over 60% of the material cost of the MFCs was due to the cation exchange membrane [40] therefore inexpensive separators such as ceramics are a valid alternative for this technology. Another important factor is the availability of the system components (electrodes, membranes, wiring etc) in order to assemble and implement the technology on a larger scale, for example one thousand and more units. The knowledge from the lab-based experiments needs to be explored in the pilot studies to assess the viability of the technology in a real environment scenario under various environmental conditions such as temperature/humidity/organic loading or pH and under operation variables such as flow rate, HRT, batch or continuous flow. This would benefit the MFC field in real-time collection of data and *in situ* knowledge. MFC reactors can degrade pollutants and generate electricity simultaneously, potentially decreasing cost, energy consumption and treatment cycle. From the energy analysis point of view, 14 Wh achieved at the last Pee Power field trial at Glastonbury 2017 [58^•^] would be the equivalent to 0.23 British pence of energy saving for every kWh. This is based on raw power data produced during the field trial where the MFC stack was operated on neat human urine and does not take into account the saving that would be gained for every litre of wastewater treated.

Further understanding of ion transport selectivity and economic membrane preparation methods are vital to enable wider employment of ion exchange membranes in technical processes for sustainable development. Further progress is needed to provide field equipment that is more robust and reliable over time [59^•^] as well as the development of novel energy storage and energy harvesting methods [10]. In energy storage, the use of external capacitors has been implemented in numerous practical applications however an integration of internal supercapacitors could be a novel way to boost and/or control the output [60].The knowledge built on the existing pilot studies and implementation attempts is driving the innovation towards wider acceptance and market.

## Conclusions

Only one type of BES can break down waste and generate electricity, and that is the MFC. Future advances should be focused on the technology applicability and the system design in order to meet the criteria of **high performance and low cost** in real-world conditions. The general trend towards the future MFC scale-up is firstly: the **reduction in size** of units but also the multiplicity of the total numbers of units, by use of **modularity,** as a way of overcoming transport limitations and ohmic losses instead of enlarging a single unit. Secondly, it is the **design of the scaled-up units (modules) through compacting the system** footprint to achieve high power densities but at the same time making it functional thus applicable in real-life scenarios. Thirdly, to ensure the **longevity** of the system and its components, both internal and external elements should be resistant to biofouling, scaling and corrosion. Finally, new developments should include MFC power management systems and the incorporation of energy harvesting and storage systems such as supercapacitors in order to enhance system performance for practical use.

## References and recommended reading

Papers of particular interest, published within the period of review, have been highlighted as:

• Paper of special interest.
•• Paper of outstanding interest.
